# Infections during AML induction chemotherapy in a contemporary cohort without fluoroquinolone prophylaxis

**DOI:** 10.1007/s15010-025-02651-4

**Published:** 2025-10-01

**Authors:** S. Ehrlich, J. Eufinger, N. Tahiri, V. Jurinovic, S. Mansournia, W. G. Kunz, J. Jung, T. Herold, M. Subklewe, V. Bücklein, M. von Bergwelt-Baildon, K. Spiekermann

**Affiliations:** 1 Department of Medicine III, University Hospital Munich - Campus Großhadern, Munich, Germany; 2 Institute for Medical Information Processing, Biometry, and Epidemiology, LMU Munich, Munich, Germany; 3 Department of Radiology, University Hospital Munich, Munich, Germany; 4 Department of Medical Microbiology and Hospital Hygiene, Max-Von-Pettenkofer Institute, LMU Munich, Munich, Germany; 5 Department of Hematology, Oncology, and Stem-Cell Transplantation, Munich Hospital Bogenhausen, Munich Municipal Hospital Group, Munich, Germany; 6 Gene Center, LMU University, Munich, Germany; 7 German Cancer Consortium (DKTK), Partner Site Munich and German Cancer Research Center (DKFZ), Heidelberg, Germany

**Keywords:** AML, Infection, invasive fungal infections, Febrile neutropenia, infection-associated mortality

## Abstract

**Purpose:**

Recent advances in the treatment of acute myeloid leukemia (AML) and optimized supportive care have improved survival outcomes. However, infections during remission induction chemotherapy remain a leading cause of morbidity and mortality. While antifungal prophylaxis is standard, the role of routine antibacterial prophylaxis is increasingly debated due to adverse effects and resistance. This study aimed to characterize infectious complications in a real-world AML cohort receiving induction chemotherapy without routine antibacterial prophylaxis.

**Methods:**

We retrospectively analyzed 103 adults with newly diagnosed AML who underwent intensive induction therapy at LMU University Hospital between January 2019 and December 2022. All patients received antifungal prophylaxis whereas antibacterial fluoroquinolone (FQ) prophylaxis was not administered. We assessed febrile episodes, clinically and microbiologically documented infections, ICU/IMC admissions, and 30-/90-day mortality.

**Results:**

Febrile episodes occurred in almost all patients. Clinically documented infections accounted for 29.8% and microbiologically confirmed infections for 22.9% of febrile events. Bacteraemia was evenly distributed between Gram-positive and Gram-negative pathogens; multidrug resistance was rare. Proven or probable invasive fungal infections occurred in 6.8% of patients. In 47.2% of cases, the cause of fever remained unknown. Infection-related 30-day mortality was 4.9%. Factors associated with increased 30-day mortality included age ≥ 65 years, ECOG ≥ 2, secondary AML, and ICU/IMC admission for infection.

**Conclusion:**

Infections remain a major challenge during AML induction therapy. Our findings suggest that FQ prophylaxis should be reevaluated in this setting, focussing on a more individualized approach. In addition, novel diagnostic tools are urgently needed to enable earlier and more targeted infection management in this high-risk population.

**Supplementary Information:**

The online version contains supplementary material available at 10.1007/s15010-025-02651-4.

## Introduction

Over the past two decades, the survival rate of patients with acute myeloid leukemia (AML) has significantly improved. Especially the approval of novel targeted therapies, such as FLT3 inhibitors and the antibody-drug conjugate gemtuzumab ozogamicin (GO), in combination with traditional cytotoxic chemotherapy, as well as the use of CPX-351 (liposomal daunorubicin and cytarabine) in patients with secondary AML (sAML) have significantly increased remission rates [1–3].

Nevertheless, infectious complications during induction therapy are frequent and contribute substantially to treatment-related morbidity and early mortality [4, 5]. Factors such as prolonged neutropenia, mucosal barrier injury, and the use of central venous catheters significantly increase the risk of bacterial and fungal infections [6, 7]. As a result, most patients experience febrile episodes during induction chemotherapy, and infection-related mortality ranges between 2 and 10% depending on cohort characteristics and treatment intensity [4, 5, 8, 9].

Emerging data suggest that the infection risk profile in AML may be changing in the context of newer therapies. For example, the broader use of GO has been associated with gastrointestinal toxicity and NEC [10], while CPX-351 is linked to delayed neutrophil count recovery which is a relevant risk factor especially for invasive fungal infections (IFIs) [11, 12]. Furthermore, a recent multicenter observational study by Cattaneo et al. reported an incidence of IFI of over 20% during induction therapy with midostaurin, despite the use of antifungal prophylaxis [13].

Understanding the infection risk is important to guide the use of anti-infecitve prophylaxis. While the benefit of antifungal prophylaxis with anti-infective posaconazole has been demonstrated clearly [14], antibiotic—most commonly with FQs—is increasingly viewed with scepticism due to potential side-effects and increasing resistance rates. Consequently, antibacterial prophylaxis for inpatients is only administered in exceptional cases at our clinic.

While contemporary AML treatment and supportive care have evolved considerably, data on infection rates during induction therapy in the era of new therapeutic options remain limited. This study therefore aims to determine the incidence, spectrum, and clinical outcomes of infections in AML patients receiving intensive remission induction chemotherapy in a contemporary cohort without FQ prophylaxis.

## Methods

### Patients and setting

We conducted a single-center retrospective cohort study at LMU University Hospital, Munich, a tertiary care center in Germany. Adults (≥ 18 years) with newly diagnosed AML who received intensive remission induction chemotherapy between January 2019 and December 2022 were eligible. Patients with acute promyelocytic Leukemia were excluded. Diagnosis was established by peripheral blood and bone marrow examinations, and cytogenetic/molecular risk stratification followed the European LeukemiaNET (ELN) 2017 criteria [15]. The observation period started on the first day of remission induction chemotherapy and continued until patients were either discharged from the hospital or the subsequent chemotherapy (e.g. consolidation) was initiated, whichever was earlier. All patients remained hospitalized during the entire induction cycle. This study was conducted in accordance with the 1964 Declaration of Helsinki and its later amendments and approved by the ethics committee of the LMU University Hospital Munich (Reference Number: 22–0528). Written informed consent for data collection, pseudonymization, and analysis was obtained from all patients through participation in the AMLCG registry.

### Chemotherapy regimens and supportive therapy

The standard induction chemotherapy regimen consisted of the 7 + 3 protocol, with or without targeted therapies. This protocol included cytarabine (200 mg/m²) as a continuous intravenous infusion on days 1–7 and daunorubicin (60 mg/m²) as a 1-hour infusion on 3 consecutive days. Other treatment regimens used were CPX-351 (liposomal daunorubicin 44 mg/m² and cytarabine 100 mg/m² on days 1, 3 and 5) and S-HAM, consisting of cytarabine at 3 g/m² (1 g/m² for patients aged > 60 years) twice daily on days 1, 2, 8, and 9, and mitoxantrone (10 mg/m²) on days 3, 4, 10, and 11 (Suppl. Table 1) [16].

Antifungal prophylaxis was initiated at the onset of severe neutropenia and continued until its resolution in all intensively treated patients (Suppl. Table 2). The choice of the antifungal agent was based on AGIHO guidelines, drug-drug interactions, and the patient’s allergy profile. Patients additionally received antiviral prophylaxis with acyclovir (400 mg BID) and/or cotrimoxazole (960 mg BID twice weekly), according to individual risk profiles and clinical judgment. Antibacterial prophylaxis and granulocyte colony-stimulating factor (G-CSF) were not routinely administered.

### Data collection, definitions, and infection assessment

The electronic medical records of patients were reviewed to collect data on demographics, AML status, chemotherapy regimens, radiological and microbiological findings, as well as overall mortality at days 30 and 90 after the initiation of the remission induction chemotherapy. Patients were monitored daily for signs of infection.

Fever was defined as a single oral temperature of ≥ 38.3 °C or a temperature of ≥ 38.0 °C lasting for more than 1 h. Neutropenia was defined as an absolute neutrophil count (ANC) < 0.5 × 10^9^/L, < 1 × 10^9^/L with a predicted decline to < 0.5 × 10^9^/L in the next 48 h, or a white blood cell count (WBC) < 1 × 10^9^/L if a differential was not available.

In cases of neutropenic fever, diagnostic workup was conducted in accordance with the national guidelines [17]. This included at least two sets of blood cultures and additional diagnostic measures depending on the suspected site of infection. Empirical antibacterial therapy was initiated immediately and typically consisted of piperacillin-tazobactam. Anti-infective treatment was subsequently adjusted if a pathogen was identified.

Infections were defined as fever requiring antibiotic therapy and/or clinical signs and symptoms associated with the isolated pathogen or an identifiable site of infection by physical examination or imaging. Infections were classified as *“clinically documented”* if patients had characteristic signs of infection without identification of a causative pathogen. A gastrointestinal tract (GIT) infection was diagnosed if fever was present along with clinical symptoms, whether or not they could be attributed to a specific pathogen.Diarrhea alone did not meet the criteria for a GI tract infection. Neutropenic enterocolitis (NEC) was defined as neutropenia < 0.5 × 10^9^/L, fever ≥ 38.3 °C, and abdominal pain with CT evidence of bowel wall thickening >4 mm >3 cm [18]. Pneumonia was diagnosed based on an abnormal chest CT scan, accompanied by clinical symptoms of a lower respiratory tract infection, including fever. Urinary tract infections (UTI) were classified as *“confirmed”* if microbiological tests showed at least 10^5^ colony-forming units per ml. *“Microbiologically documented”* infections were defined as infections presenting with characteristic symptoms and concurrent evidence of a relevant pathogen. Infections were categorized as bacterial, viral, fungal, or fever of unknown origin (FUO). Viral isolates such as EBV *(Epstein-Barr virus)* and HSV *(herpes simplex virus)* without clinical symptoms were not considered causative for a febrile episode, as these were likely reactivations associated with immunosuppression. If multiple pathogens of the same type were identified in a single sample or at another site, it was considered a single infection. Bacterial isolates were excluded from the analysis if they were obtained from only one blood culture and were highly likely to be skin contaminants. IFIs were diagnosed according to the European Organization for Research and Treatment of Cancer (EORTC) and Mycoses Study Group Education and Research Consortium (MSGERC) guidelines and classified as “*proven”*, “*probable”*, or “*possible”* [12]. Breakthrough mycoses were categorized based on the MSGERC/ECMM consensus definitions [19].

### Statistical analysis

Descriptive statistics were applied for baseline and infection data. Univariate logistic regression was used to assess associations with 30-day mortality, reported as odds ratio (OR) with 95% confidence intervals (CI). Multivariate analysis was not performed due to the limited number of events. All statistical analyses were conducted using GraphPad Prism version 10.4.0 (GraphPad Software, Boston, USA).

## Results

### Patient characteristics and treatment

A total of 103 patients with newly diagnosed AML were included in this retrospective analysis. Baseline characteristics and treatment details are summarized in Table 1. The median age was 58 years, and 45.6% of patients were female. The majority of patients presented with de novo AML. The distribution across ELN 2017 risk categories was relatively balanced, with a slight underrepresentation in the intermediate-risk group.

**Table 1:** Baseline characteristics (first day of remission induction chemotherapy)

	*N* = 103
Age in years, Median (Range)	58 (19–77)
Female sex, *n* (%)	47 (45.6)
AML subgroup	
De novo AML, *n* (%)	88 (85.4)
Secondary AML, *n* (%)	15 (14.6)
ELN 2017	
Favorable, *n* (%)	38 (36.9)
Intermediate, *n* (%)	29 (28.2)
Adverse, *n* (%)	35 (34.0)
Na, *n* (%)	1
ECOG	
< 2, *n* (%)	86 (83.5)
≥ 2, *n* (%)	17 (16.5)
BMI, Median (Range)	24.6 (16.7–39.3)
Laboratory results	
WBC, x10^9^/L, Median (Range)	6.84 (0.11–355)
ANC, x10^9^/L, Median (Range)	0.71 (0.1–19.2)
ANC < 0.5 × 10^9^/L, Median (%)	43 (41.7)
CRP, mg/L, Median (Range)	24 (1-519)
Antibiotic therapy, *n* (%)	57 (55.3)

*AML* acute myeloid leukemia, *ELN* European LeukemiaNET classification, *ECOG* Eastern Cooperative Oncology Group performance status, *BMI* body mass index, *WBC* white blood count, *ANC* absolute neutrophil count, *CRP* C-reactive protein

At diagnosis, most patients exhibited good performance status (ECOG < 2), and the median body mass index (BMI) was 24.6 kg/m². On the day of induction, the median leukocyte count was 6.84 × 10⁹/L and the median absolute neutrophil count (ANC) was 0.71 × 10⁹/L. Nearly half of the patients (41.7%) presented with severe neutropenia (ANC < 0.5 × 10⁹/L) at baseline.

Induction regimens included “7 + 3” (28.2%), “7 + 3” in combination with midostaurin (38.8%) or GO (15.5%) and CPX-351 (8.7%) (Suppl. Table 1). Six patients received two cycles of induction chemotherapy. All but one patient received antifungal prophylaxis, primarily with posaconazole (79.6%) or echinocandins (18.4%) (Suppl. Table 2). On the first day of induction, 55.3% of patients had an ongoing antibiotic therapy. Most common causes for antibiotic therapy were FUO (27.2%) and pneumonia (14.6%) (Suppl. Table 3).

### Febrile episodes

**Fig. 1:**
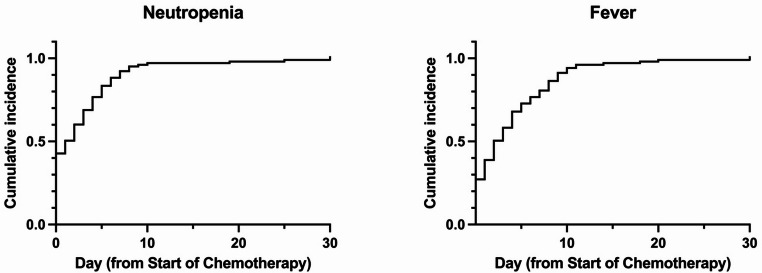
Cumulative incidence probability of neutropenia (**A**) and fever (**B**) over 30 days from the initiation of chemotherapy

Fever was observed in 51.5% of patients prior to induction (Table 2). Post-induction neutropenia occurred in 99.0% of patients, with a median duration of 22 days. Fever developed in 99.0% of patients, totaling 218 febrile episodes. In 97.1% of the cases, fever occurred during periods of severe neutropenia. Both fever and neutropenia typically emerged within the first 10 days of chemotherapy initiation (Fig. 1). Approximately half of all febrile episodes remained of unknown origin.

**Table 2:** Fever and infection

	*N* = 103
Fever prior to induction chemotherapy (% of patients)	53 (51.5)
Neutropenia after induction chemotherapy (% of patients)	102 (99.0)
Neutropenia (ANC < 0.5 × 10^9^/L) in days, Median (Range)	22 (1–92)
Febrile episodes	218
≥ 1 episode of fever (% of patients)	102 (99.0)
≥ 1 episode of febrile neutropenia (% of patients)	100 (97.1)
Clinically documented infection (% of febrile events)	65 (29.8)
Microbiologically documented infection (% of febrile events)	50 (22.9)
Bacterial (% of febrile events)	41 (18.8)
Fungal (% of febrile events)	7 (3.2)
Viral (% of febrile events)	2 (0.9)
FUO (% of febrile events)	103 (47.2)

*IFI* invasive fungal infection, *ANC* absolute neutrophil count, *FUO* fever of unknown origin

### Clinically documented infections

**Fig. 2:**
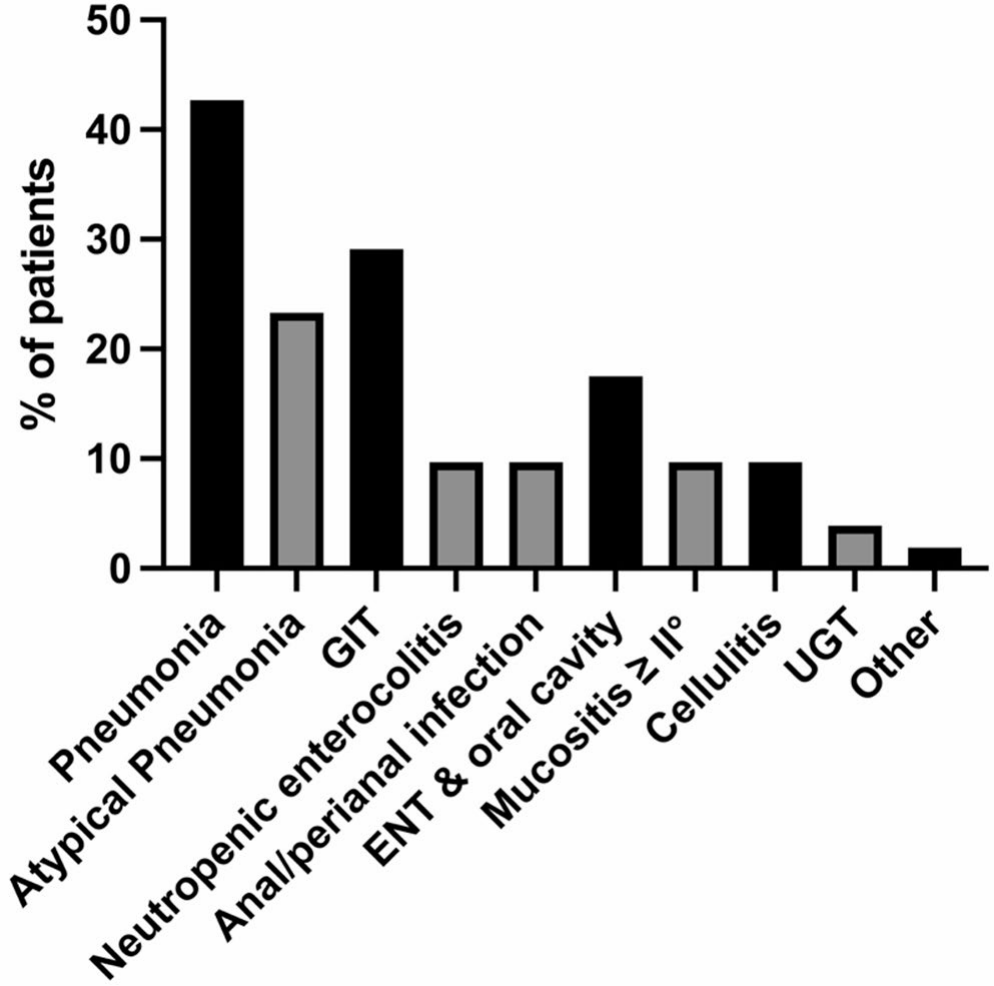
Clinically documented infections. Data are shown in % of patients. Different infections in a single patient were counted separately. Black bars indicate the main clinically documented infections while grey bars give more detailed information about the site of infection *GIT* gastrointestinal tract, *ENT* ear nose & throat, *UGT* urogenital tract

Among all febrile episodes, 29.8% were classified as clinically documented infections. In total, 139 infectious foci were identified in 84.2% of patients, with some individuals presenting with multiple sites of infection either simultaneously or at different time points (Fig. 2). Pneumonia was the most frequent clinical infection (42.7%), including atypical pneumonia in 23.3% of patients. GIT infections were the second most common infection (29.1%), with 10 cases meeting the criteria for neutropenic enterocolitis and 10 additional cases presenting with a perianal focus. Ear, nose and throat infections were documented in 17.5% of patients, most frequently due to mucositis grade ≥ II. Furthermore, cellulitis was observed in 11.7% and urinary tract infections in 3.9%.

### Microbiologically documented infections

Microbiologically documented infections accounted for 22.9% of all febrile episodes (Table 2). Bacterial infections typically occurred within three weeks after induction chemotherapy, whereas half of the IFIs were detected after more than four weeks (Suppl. Figure 1). A total of 55 such infections were identified in 40 patients (38.8%), the majority of which were bacterial in origin. Importantly, five individuals had different microbiologically documented infections at the same timepoint. An overview of bloodstream pathogens is provided in Table 3, while other microbiologically confirmed infections are detailed in Suppl. Table 4.

**Table 3:** Distribution of isolates in BSI (bloodstream infection)

	*N* (%)
Patients with BSI	29 (28.2)
Patients with CRBSI	5 (4.9)
Patients with > 1 BSI	5 (4.9)
Patients with polymicrobial bloodstream infection	4 (3.9)
Isolates from blood stream infections	37
Gram-positive (% of isolates)	17 (45.9)
Staphylococci	6
Coagulase negative	3
Linezolid-resistant *Staphylococcus epidermidis*	1
*Staphylococcus aureus*	2
Streptococci	2
Enterococci	8
*Enterococcus faecium*	4
Vancomycin-resistant *Enterococcus faecium*	4
*Corynebacterium amycolatum*	1
Gram-negative (% of isolates)	17 (45.9)
Enterobacteriaceae	11
*Escherichia coli*	9
*Klebsiella spp.*	1
*Enterobacter cloacae*	1
Non-fermenting	6
*Pseudomonas aeruginosa*	4
*Stenotrophomonas maltophilia*	2
Fungi (% of isolates)	3 (8.2)
*Candida glabrata* * (Nakaseomyces glabratus)*	1
*Saprochaete capitata*	1
*Fusarium solani*	1

*Spp.* Species, *BSI* bloodstream infection

Bloodstream infections occurred in 28.2% of patients (*n* = 29), including five catheter-associated infections. Five patients developed multiple episodes, and four had polymicrobial infections. In total, 37 bloodstream isolates were analyzed. Gram-positive and Gram-negative bacteria each accounted for 45.9% of the cases, while fungi were responsible for 8.2%. Among Gram-positive organisms, coagulase-negative staphylococci, *Staphylococcus aureus*, and enterococci were most frequently detected, including four vancomycin-resistant *Enterococcus faecium* strains. Gram-negative isolates included *Escherichia coli*, *Klebsiella spp.*, *Enterobacter cloacae*, *and Pseudomonas aeruginosa*.

According to EORTC/MSG criteria, seven patients (6.8%) met the definition of proven or probable IFI (Table 4). Proven IFIs were diagnosed via blood cultures and included *Saprochaete capitata*, *Fusarium solani*, and *Candida glabrata (Nakaseomyces glabratus)*. In one patient, *Fusarium solani* and *Pneumocystis jirovecii* were identified at different time points. Three patients had probable pulmonary aspergillosis. All but one IFI fulfilled the criteria for breakthrough infection; notably, *Saprochaete capitata* was resistant to caspofungin, which had been administered as prophylaxis. In 16.5% of all patients, a possible pulmonary IFI was diagnosed.

**Table 4:** Distribution of probable and proven invasive fungal infections

	*N* = 103 (%)	Site of Detection
Proven or probable IFI	7 (6.8)	
*Aspergillus spp.*	3 (2.9)	Lung
*Fusarium solani*	1 (1.0)	Blood
*Candida glabrata (Nakaseomyces glabratus)*	1 (1.0)	Blood
*Saprochaete capitata*	1 (1.0)	Blood
*Pneumocystis jirovecii*	1 (1.0)	Lung
*Rhizopus microsporus*	1 (1.0)	Rhinocerebral
Possible IFI	17 (16.5)	Lung

*Spp.* Species, *IFI* invasive fungal infection

Only one febrile episode was attributable to a viral infection, namely Influenza A. Other viral isolates, such as HSV and EBV detected in throat washes, were interpreted as viral reactivations in the context of immunosuppression (Suppl. Table 5). We did not observe any COVID-19 infections.

### ICU admissions and infection-related mortality

During the observation period, 21.4% of patients (*n* = 22) required intensive care unit (ICU) admission, with more than half of these cases attributed to infection (Table 5). Causes included sepsis (*n* = 5), IFI (*n* = 3), pneumonia (*n* = 2), and other infections (*n* = 3). Additionally, 16.5% of patients (*n* = 17) were transferred to intermediate care (IMC), with infections accounting for 10 out of 17 of transfers.

**Table 5:** Outcome

	*N* = 103
ICU, *n* (%)	22 (21.4)
Due to infection, *n* (%)	13 (12.6)
Sepsis	5 (4.9)
IFI	3 (2.9)
Pneumonia	2 (1.9)
Other	3 (2.9)
IMC, *n* (%)	17 (16.5)
Due to infection, *n* (%)	10 (9.7)
Remission status	
CR, *n* (%)	58 (56.3)
na, *n* (%)	4 (3.9)
30-day mortality	8 (7.8)
Infection-associated mortality at day 30	5 (4.9)
90-day mortality	10 (9.7)
Infection-associated mortality at day 90	6 (5.8)

*ICU* intensive care unit, *IMC* intermediate care unit, *CR* complete remission, *IFI* invasive fungal infection

30-day overall mortality was 7.8%, with infection-related deaths comprising 4.9% (63%). At 90 days, overall mortality increased to 9.7%, with 5.8% (60%) of deaths attributed to infections. Detailed causes of death are listed in Suppl. Table 6.

### Risk factors for early mortality and severe infections

Univariate logistic regression identified several factors significantly associated with 30-day mortality (Table 6), including age ≥ 65 years (OR 5.53, 95 % CI 1.22–25.00; *p* = 0.026), ECOG performance status ≥ 2 (OR 6.92, 95 % CI 1.52–31.38; p = 0.012), secondary AML (OR 7.64, 95 % CI 1.67–34.98; p = 0.009), and severe infections requiring intermediate or intensive care treatment (OR 12.72, 95 % CI 2.67–60.76; p = 0.012). No statistically significant associations were observed for ELN 2017 risk classification, microbiologically documented infections, or the presence of IFI.

**Table 6:** Risk factors for 30-day mortality. Univariate logistic regression

Variable	Comparison	OR (95% CI)	*p*-value
Age	≥ 65 vs. <65	5.53 (1.22–25.00)	0.026
ECOG	≥ 2 vs. <2	6.92 (1.52–31.38)	0.012
ELN 2017	Adverse vs. favorable & intermediate	2.75 (0.58–13.06)	0.202
Diagnosis	sAML vs. de novo AML	7.64 (1.67–34.98)	0.009
Microbiologically documented infection	Yes vs. No	1.89 (0.44–8.00)	0.394
Severe infection (Treatment on IMC/ICU)	Yes vs. No	12.72 (2.67–60.76)	0.012

*ELN* European LeukemiaNET classification, *AML* acute myeloid leukemia, *ECOG* Eastern Cooperative Oncology Group performance status, *IMC* intermediate care, *ICU* intensive care unit

Factors associated with severe infections necessitating ICU or IMC treatment included fever prior to induction chemotherapy, elevated baseline C-reactive protein (CRP), neutropenic enterocolitis (NEC), and IFI. The addition of targeted therapies did not significantly influence the risk of severe infection (Suppl. Table 7). Multivariate logistic regression was not performed due to the limited number of events, which precluded a reliable model.

## Discussion

This retrospective single-center study of 103 patients with newly diagnosed AML provides insights into the incidence, spectrum, and clinical impact of infectious complications during remission induction chemotherapy in a setting without antibacterial prophylaxis.

The most frequent clinically documented infection was pneumonia, including a notable proportion of atypical pneumonia. The high rate of pneumonia (42.7%), particularly atypical forms, reflects the profound immunosuppression during induction therapy, but may also be the result of frequently performed chest CT scans in case of persisting fever. Comparable studies report incidences between 20% and 51% [4, 8, 20, 21]. The rate of proven and probable IFI (6.8%) observed is comparable to recent real-world data [22, 23], but much lower than in earlier reports [5, 24, 25], most likely reflecting the consistent use of antifungal prophylaxis.

BSIs were documented in 28.2% of patients—comparable to rates reported in cohorts receiving antibacterial prophylaxis [4, 21, 26]. The relatively high rate of Gram-negative isolates may reflect the absence of FQ prophylaxis. Previous studies have shown a shift toward Gram-positive organisms in cancer patients with neutropenia [27]. In line with other studies not using FQ prophylaxis, we observed low rates of multidrug-resistant organisms [28].

Interestingly, no COVID-19 infections were observed despite frequent testing, which is likely attributable to comprehensive screening procedures and strict isolation measures for infected individuals and high-risk patients.

Approximately one-third of patients experienced GIT infections, including 10% with neutropenic enterocolitis (NEC). These findings are consistent with prior studies [29, 30]. Risk factors include the use of cytotoxic agents such as cytarabine, prolonged neutropenia and pre-existing bowel abnormalities [31], but as recently demonstrated, also the administration of GO [10]. Reported incidences vary considerably, largely due to differences in patient populations and diagnostic criteria. A recent systematic review estimated the incidence of NEC at 5.3% in adults with acute leukemia [30], indicating the need for further evaluation in prospective studies.

Despite extensive diagnostic efforts, a large proportion of febrile episodes remained classified as FUO, a well-known phenomenon in intensively treated patients [5, 32, 33]. Due to the lack of specific clinical or laboratory markers to differentiate infectious from non-infectious fever in neutropenia, empirical broad-spectrum antibiotic therapy remains standard. In our cohort, 16.5% of patients also met the criteria for possible pulmonary aspergillosis. While these criteria lack specificity, they frequently prompt empirical antifungal treatment, potentially leading to overtreatment. This underscores the need for improved diagnostics such as standardized use of PCR or next-generation sequencing (NGS)-based approaches, which may enhance early detection in high-risk patients. Especially NGS-based approaches show promise for infection detection in immunocompromised patients [34], but their clinical utility remains to be established in future studies.

Infections accounted for 59% of ICU admissions in our cohort, a rate comparable to studies employing antibacterial prophylaxis [8, 21]. Infection-related mortality within 30 days was 4.9%, with IFIs being the cause of death in two of five cases. These findings must be interpreted considering cohort heterogeneity, which included patients receiving CPX-351 or S-HAM—both associated with prolonged neutropenia [8, 21]. Overall, our results align with a recent meta-analysis by Owattanapanich and Chayakulkeeree, which demonstrated that FQ prophylaxis reduces the incidence of febrile episodes and BSIs but does not significantly impact mortality [35].

Risk factor analysis identified age ≥ 65 years, ECOG performance status ≥ 2, sAML, and infections requiring ICU or intermediate care as significant predictors of 30-day mortality, consistent with previous data [36]. Notably, the presence of microbiologically documented infections alone was not associated with increased mortality, emphasizing the critical role of infection severity and clinical management. Remission status could not be assessed as a prognostic factor due to missing response data in patients who died within 30 days. The sample size and low event rate did not allow for multivariate regression analysis.

Another finding of our study is the high rate of febrile episodes post-induction chemotherapy, occurring in nearly all patients (99.0%). This is in line with previously reported incidences, with incidences ranging from 60 to 93% depending on study design and treatment protocols with generally higher incidences in cohorts without antibacterial prophylaxis [4, 5, 8, 21, 26, 37]. However, direct comparison is limited due to heterogeneity in study design, definitions, and treatment protocols.

Compared to cohorts with FQ prophylaxis, the higher incidence of neutropenic fever may have led to an increased use of broad-spectrum antibiotics. However, FQ use has been linked with rare but severe side effects, such as tendinopathy or aortic dissection. In response, regulatory authorities in Europe have substantially restricted their approved indications since 2018 [38]. Furthermore, administration of FQ-prophylaxis is associated with higher rates of colonization and infections with FQ-resistant bacteria in AML-patients [27, 39], complicating infection management. Furthermore, it does not improve overall mortality [35]. Nevertheless, it remains recommended for high-risk patients when the goal is to reduce the incidence of fever and BSIs [40]. Considering the potential risk of FQ prophylaxis, a future approach could involve restricting antibacterial prophylaxis to patients with identified risk factors like sAML, age and ECOG. While our data suggest that selected patients can be managed safely without FQ prophylaxis, these results should be interpreted with caution as they are based on a retrospective single-center study and on univariate rather than multivariate analyses. The small number of events precluded multivariate adjustment, which represents a key limitation of our work. Moreover, most patients had already received systemic antibiotic therapy at the time of induction, which may have influenced both baseline infection status and the subsequent incidence and spectrum of infectious complications. Future studies should provide further data on the long-term efficacy of prophylaxis versus empirical therapy to support evidence-based decision-making.

This study has several other limitations. Its retrospective nature introduces potential bias due to incomplete documentation. Furthermore, the single-center design and heterogeneity in induction regimens limit generalizability. Nonetheless, the study provides relevant real-world data on infection patterns in AML patients treated without antibacterial prophylaxis.

In summary, our findings demonstrate that infections remain a major cause of early morbidity and mortality during AML induction therapy. Despite novel treatment options and advances in supportive care, infectious complications continue to represent a substantial clinical challenge. Future studies should reassess the role of FQ prophylaxis focussing on a more individualized approach. Furthermore, our data show that novel diagnostic approaches are urgently needed to enable earlier and more targeted detection and treatment of infections.

## Supplementary Information

Below is the link to the electronic supplementary material.

Supplementary Material 1

## Data Availability

All original data will be made available upon reasonable request to the corresponding author.
