# Correction: Infections during AML induction chemotherapy in a contemporary cohort without fluoroquinolone prophylaxis

**DOI:** 10.1007/s15010-025-02705-7

**Published:** 2025-12-22

**Authors:** S. Ehrlich, J. Eufinger, N. Tahiri, V. Jurinovic, S. Mansournia, W. G. Kunz, J. Jung, T. Herold, M. Subklewe, V. Bücklein, M. von Bergwelt-Baildon, K. Spiekermann

**Affiliations:** 1 Department of Medicine III, University Hospital Munich - Campus Großhadern, Munich, Germany; 2 Institute for Medical Information Processing, Biometry, and Epidemiology, LMU Munich, Munich, Germany; 3 Department of Radiology, University Hospital Munich, Munich, Germany; 4 Department of Medical Microbiology and Hospital Hygiene, Max-Von-Pettenkofer Institute, LMU Munich, Munich, Germany; 5 Department of Hematology, Oncology, and Stem-Cell Transplantation, Munich Hospital Bogenhausen, Munich Municipal Hospital Group, Munich, Germany; 6 Gene Center, LMU University, Munich, Germany; 7 German Cancer Consortium (DKTK), Partner Site Munich and German Cancer Research Center (DKFZ), Heidelberg, Germany

**Correction: Infection** 10.1007/s15010-025-02651-4

The original text stated logistic regression was performed, while the original Table 6 showed hazard ratios stemming from an outdated version. Table 6 has been updated to correctly display the results from the Logistic Regression analysis.

**Table 6:** Risk factors for 30-day mortality. Univariate logistic regression

Variable	Comparison	OR (95% CI)	p-value
Age	≥ 65 vs. < 65	5.53 (1.22–25.00)	0.026
ECOG	≥ 2 vs. < 2	6.92 (1.52–31.38)	0.012
ELN 2017	Adverse vs. favorable & intermediate	2.75 (0.58–13.06)	0.202
Diagnosis	sAML vs. de novo AML	7.64 (1.67–34.98)	0.009
Microbiologically documented infection	Yes vs. no	1.89 (0.44–8.00)	0.394
Severe infection (treatment on IMC/ICU)	Yes vs. no	12.72 (2.67–60.76)	0.012

*ELN* European LeukemiaNET classification, *AML* acute myeloid leukemia, *ECOG* Eastern Cooperative Oncology Group performance status, *IMC* intermediate care, *ICU* intensive care unit

The corresponding sentence in the section ‘Risk factors for early mortality and severe infections’ has been updated accordingly.

Table 3 has been revised to enhance clarity by providing a distinct differentiation between percentages calculated as ‘% of patients’ and ‘% of isolates’, which was previously ambiguous.

**Table 3:** Distribution of isolates in BSI

	N (%)
Patients with BSI	29 (28.2)
Patients with CRBSI	5 (4.9)
Patients with > 1 BSI	5 (4.9)
Patients with polymicrobial bloodstream infection	4 (3.9)
Isolates from blood stream infections	37
Gram-positive (% of isolates)	17 (45.9)
Staphylococci	6
Coagulase negative	3
Linezolid-resistant *Staphylococcus epidermidis*	1
*Staphylococcus aureus*	2
Streptococci	2
Enterococci	8
*Enterococcus faecium*	4
Vancomycin-resistant *Enterococcus faecium*	4
*Corynebacterium amycolatum*	1
Gram-negative (% of isolates)	17 (45.9)
Enterobacteriaceae	11
*Escherichia coli*	9
*Klebsiella *spp.	1
*Enterobacter cloacae*	1
Non-fermenting	6
* Pseudomonas aeruginosa*	4
* Stenotrophomonas maltophilia*	2
Fungi (% of isolates)	3 (8.2)
*Candida glabrata (Nakaseomyces glabratus)*	1
*Saprochaete capitata*	1
*Fusarium solani*	1

*Spp.* Species, *BSI* bloodstream infection

We detected and corrected typographical errors: Removal of the unintentionally inserted text ‘Write “AML”’ in the section ‘Patients and Setting’, Table 2 (‘10^3^/L’ to ‘10^9^’), Table 5 (‘58.3%’ to ‘56.3%’).

We confirm that the scientific conclusions remain unchanged. The original article has been updated.

