# Supplementary material for: Infections during AML induction chemotherapy in a contemporary cohort without fluoroquinolone prophylaxis
Source: Infection. 2025 Oct 1;54(1):243–52. doi: 10.1007/s15010-025-02651-4 (PMC12864249; doi:10.1007/s15010-025-02651-4)

**Supplementary Tables and Figures**

**Suppl. Table 1** Chemotherapy regimens

| **Chemotherapy regimens** | **N (%)** |
| --- | --- |
| 7+3 (cytarabin +daunorubicin) | 29 (28.2) |
| + Midostaurin | 40 (38.8) |
| + GO | 16 (15.5) |
| + Other drug | 2 (1.9) |
| S-HAM | 7 (6.9) |
| CPX-351 (cytarabine +liposomal doxorubicin) | 9 (8.7) |

Data are presented as no. (%) unless otherwise specified.

*S-HAM* sequential high dose cytarabine and mitoxantrone, *GO* Gemtuzumab-Ozogamizin

**Suppl. Table 2** Antifungal prophylaxis

| **Drug** | **N (%)** |
| --- | --- |
| Posaconazole, n (%) | 82 (79.6) |
| Micafungin, n (%) | 12 (11.7) |
| Caspofungin | 7 (6.9) |
| Other | 1 (1.0) |

**Suppl. Table 3** Infections prior to induction chemotherapy

| **Infection** | **N (%)** |
| --- | --- |
| Pneumonia, n (%) | 15 (14.6) |
| Atypical pneumonia, n (%) | 11 (10.7) |
| ENT and oral cavity | 6 (5.8) |
| Skin & soft tissue | 4 (3.9) |
| BSI | 2 (1.9) |
| Covid-19 infection | 1 (1.0) |
| FUO | 28 (27.2) |

*ENT* ear, nose & throat, *BSI* blood stream infection, *FUO* fever of unknown origin

**Suppl. Table 4** Microbiologically documented infections

|  | **N (%)** |
| --- | --- |
| Pneumonia | 6 (5.8) |
| Bacterial |  |
| *Stenotrophomonas maltophilia* | 1 (1.0) |
| Fungal |  |
| *Aspergillus spp.* | 3 (2.9) |
| *Pneumocystis jirovecii* | 1 (1.0) |
| Viral |  |
| *Influenza A* | 1 (1.0) |
| GIT | 6 (5.8) |
| Bacterial |  |
| *Clostridium difficile* | 6 (5.8) |
| ENT & Oral Cavity | 3 (2.9) |
| Bacterial |  |
| *Actinomyces spp.* | 1 (1.0) |
| Fungal |  |
| *Rhiizopus microsporus* | 1 (1.0) |
| Viral |  |
| *HSV* | 1 (1.0) |
| Skin/Soft Tissue | 1 (1.0) |
| Bacterial |  |
| *Staphylococcus aureus* | 1 (1.0) |
| UGT | 4 (3.9)^b^ |
| Bacterial |  |
| *Escheria coli* | 2 (1.9) |
| *Enterococcus faecium* | 2 (1.9) |
| *Enterococcus faecalis* | 1 (1.0) |
| Other | 1 (1.0) |
| Viral |  |
| *HHV6* | 1 (1.0) |

^a^one patient with polymicrobial infection

^b^two patients with polymicrobial infection

*GIT* gastrointestinal tract*, ENT* ear, nose & throat, *UGT* urogenital tract, *spp.* species, *HSV* Herpes simplex virus, *HHV6* Human herpes virus 6

**Suppl. Table 5** Distribution of viral isolates

| **Viral Isolates** | **N=103** |
| --- | --- |
| No of patients (%) | 40 (35.0) |
| Isolates | 67 |
| HSV | 21 |
| HHV6 | 3 |
| EBV | 14 |
| Influenza A | 1 |
| HBV | 1 |

Data are presented as no. unless otherwise specified.

*HSV* Herpes simplex virus, *HHV6* Human herpes virus 6, *EBV* Epstein-Bar Virus, *HBV* Hepatitis B virus

**Suppl. Table 6** Cause of death

| **Day** | **ICU/IMC** | **Cause of death** |
| --- | --- | --- |
| 2 | ICU | Hyperleucocytosis |
| 3 | ICU | Septic shock due to enterocolitis |
| 5 | ICU | Progress, BSC |
| 13 | ICU | Pneumonia |
| 20 | ICU | IFD/Rhinocerebral mucormycosis |
| 23 | ICU | Cardiogenic shock (Anthracycline-associated) |
| 27 | IMC | Septic shock |
| 27 | ICU | IFI/Fungaemia (*Saprochaete capitata)* |
| 48 | IMC | Candidaemia |
| 65 | - | Progress, BSC |

*ICU* intensive care unit, *IMC* intermediate care unit, *BSC* best supportive care, *IFI* invasive fungal infection

**Suppl. Table 7** Risk factors for severe infection. Univariate logistic regression.

| **Variable** | **Comparison** | **OR (95 % CI)** | **p-value** |
| --- | --- | --- | --- |
| Age | ≥65 vs. <65 | 1.34  (0.39-4.15) | 0.6190 |
| ECOG | ≥2 vs. <2 | 2.08  (0.52-7.18) | 0.2630 |
| ELN 2017 | adverse vs. favorable & intermediate | 2.97  (0.00-2.21) | 0.0504 |
| Diagnosis | sAML vs. de novo AML | 2.30  (0.57-8.06) | 0.2263 |
| **Fever prior to induction chemotherapy** | **Yes vs. No** | **5.09**  **(1.51-23.35)** | **0.0160** |
| **CRP** | **Continuous** | **1.06**  **(1.00-1.14)** | **0.0386** |
| Microbiologically documented infection | Yes vs. No | 1.64  (0.55-4.86) | 0.3676 |
| Clinically documented infection | Yes vs. No | 0.90  (0.28-3.48) | 0.8612 |
| **Neutropenic enterocolitis** | **Yes vs. No** | **7.46**  **(1.82-31.13)** | **0.0046** |
| **IFI** | **Yes vs. No** | **9.22**  **(1.83-51.90)** | **0.0070** |
| Additional targeted therapy | Yes vs. No | 1.87  (0.62-6.36) | 0.2801 |
| Midostaurin | Yes vs. No | 1,64  (0.55-4.86) | 0.3676 |
| GO | Yes vs. No | 0.69  (0.10-2.81) | 0.6403 |
| CPX-351 | Yes vs. No | 0.66  (0.03-3.99) | 0.7033 |
| CR not achieved | Yes vs. No | 1.95  (0.64-6.08) | 0.2356 |

*ECOG* Eastern Cooperative Oncology Group, *ELN* European Leukemia Network, *CRP* C-reactive protein, *IFI* invasive fungal infection, *GO* Gemtuzumab-Ozogamicin, *CR* complete remission

**Suppl. Figure 1** Cumulative Incidence Probability of bacterial and fungal infections over 60 days from start of chemotherapy


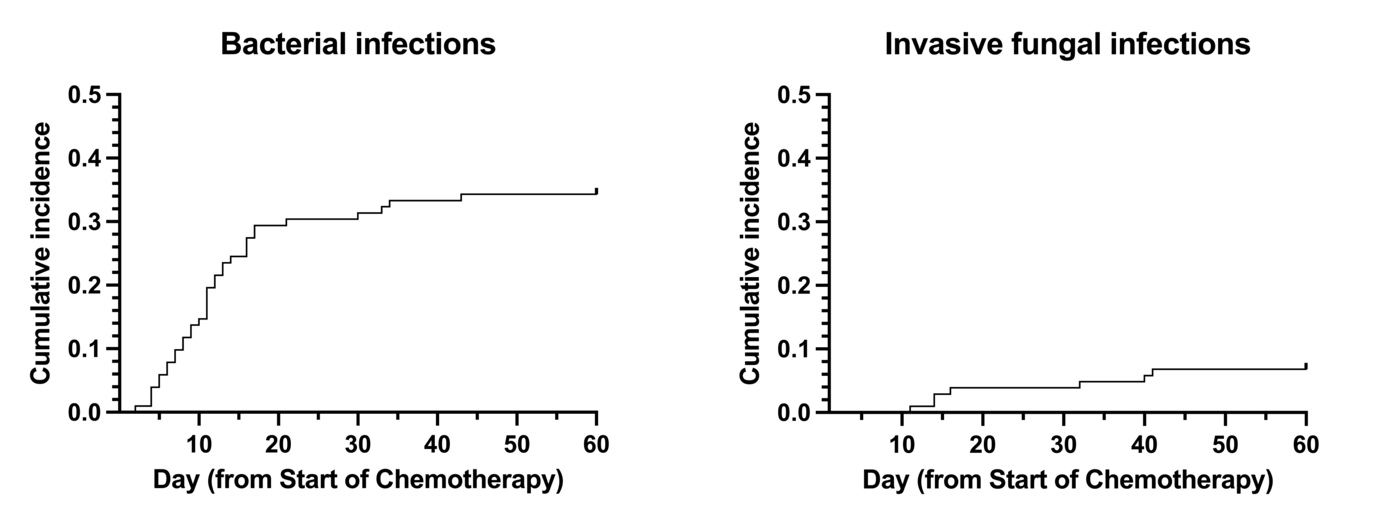

Supplement: Supplementary file 1 — Supplementary Material 1 [file 15010_2025_2651_MOESM1_ESM.docx]
